# Compound Heterozygosis in AADC Deficiency and Its Complex Phenotype in Terms of AADC Protein Population

**DOI:** 10.3390/ijms231911238

**Published:** 2022-09-23

**Authors:** Giovanni Bisello, Mariarita Bertoldi

**Affiliations:** Department of Neuroscience, Biomedicine and Movement Sciences, Section of Biological Chemistry, University of Verona, Strada le Grazie 8, 37134 Verona, Italy

**Keywords:** aromatic amino acid deficiency, homodimer, heterodimer, interallelic complementation, structure-function relationship, severity prediction

## Abstract

Aromatic amino acid decarboxylase (AADC) deficiency is a rare monogenic disease due to mutations in the *ddc* gene producing AADC, a homodimeric pyridoxal 5′-phosphate-dependent enzyme. The disorder is often fatal in the first decade and is characterized by profound motor impairments and developmental delay. In the last two years, there has been a net rise in the number of patients and variants identified, maybe also pushed by the ongoing gene therapy trials. The majority of the identified genotypes are compound heterozygous (about 70%). Efforts are underway to reach early diagnosis, find possible new markers/new fast methods, and predict clinical outcome. However, no clear correlation of genotype-to-phenotype exists to date. Nevertheless, for homozygous patients, reliable results have been obtained using genetic methods combined with available computational tools on crystal structures corroborated by biochemical investigations on recombinant homodimeric AADC variants that have been obtained and characterized in solution. For these variants, the molecular basis for the defect has been suggested and validated, since it correlates quite well with mildness/severity of the homozygous phenotype. Instead, prediction for compound heterozygous patients is more difficult since complementation effects could happen. Here, by analyzing the existing literature on compound heterozygosity in AADC deficiency and other genetic disorders, we highlight that, in order to assess pathogenicity, the measurement of activity of the AADC heterodimeric variant should be integrated by bioinformatic, structural, and functional data on the whole protein constellation theoretically present in such patients. A wider discussion on symptomatic heterozygosity in AADC deficiency is also presented.

## 1. Introduction

Aromatic amino acid decarboxylase (AADC) deficiency is a rare neurometabolic disease of monogenic inheritance leading to neurotransmitter imbalance and related severe motor and neurodevelopmental symptoms, such as hypokinesia, hypotonia, hypertonia, oculogyric crises (the hallmark of this disorder), developmental delay, and dystonia, as well as gastrointestinal and sleep problems. The first patients were described about thirty years ago [1]. In the presence of typical symptoms, the diagnosis of the disease is based on both the analysis of the cerebrospinal fluid (CSF) for metabolites related to AADC misfunctioning with a focus on the level of 3-*O*-methyldopa (3OMD), the byproduct of the accumulated substrate L-Dopa, and the determination of AADC activity in plasma [2]. This screening could then be followed and validated by genomic analyses [2]. However, as also underlined by the guidelines [2], diagnosis presents some weaknesses since the values of biogenic amines in CSF are overlapped in mild, moderate, and severe cases, and thus, they cannot be associated with the clinical phenotype. In addition, plasma AADC activity does not seem to distinguish clinical outputs since it is below the detection limit in many cases, irrespective of whether they are mild or severe [2]. Thus, at least in the less severe cases and in healthy carrier heterozygotes, diagnosis could be masked by other factors such as psychiatric symptoms due to serotonin imbalance.

The accepted clinical treatments foresee the supplementation with a drug combination: (i) vitamin B6, the precursor of the pyridoxal 5′-phosphate (PLP), the cofactor essential for AADC activity; (ii) a dopamine agonist, to supply the paucity of endogenous dopamine; iii) an inhibitor of monoamine oxidase to maintain the content of aromatic amines as high as possible (an in-depth analysis on clinical treatments is provided by other papers [2,3]).

**Figure 1:**
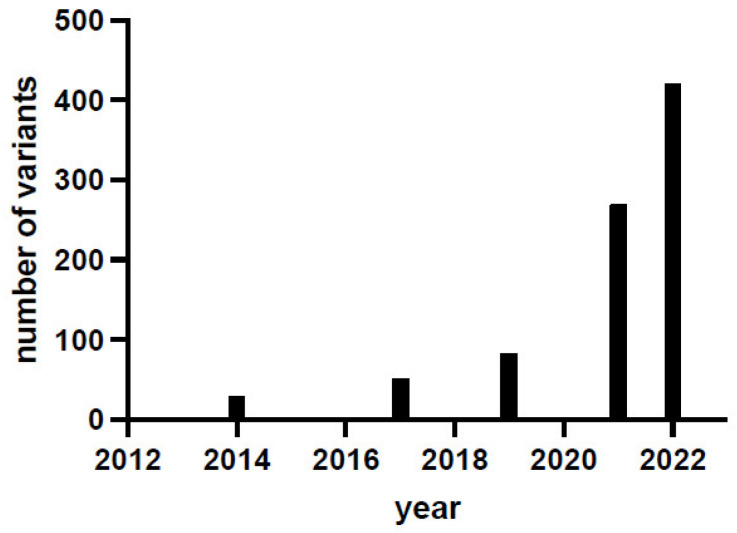
AADC deficiency variant identification in the last decade. Data are taken from [2,3,14] and from the locus-specific database PNDdb (http://www.biopku.org/pnddb/search-results.asp accessed on 30 August 2022) at the end of December 2021 and of August 2022.

The last systematic collection of genetic and clinical data was published in 2019 and is based on 123 confirmed patients and 82 variants (71% missense, 11% splice, 11% insertions/deletions/duplications, 7% others) in 58 genotypes (33% in homozygosis and 67% in compound heterozygosis), and no clear genotype-to-phenotype correlation has been established [3]. In the last three years, there has been a marked increase in the number of identified variants, as shown by the locus-specific database PNDdb (http://biopku.org/home/pndbdb.asp accessed on 30 August 2022) that currently (August 2022) reports 420 variants, highlighting that the number of variants has exponentially increased (Figure 1), with the compound heterozygous genotype contributing over 70% of genotypes [4]. The clinical trials of gene therapy based on adeno-associated virus delivered either to the putamen [5,6,7,8,9,10] or to the midbrain [11,12] could have boosted the interest in this rare disease. Gene therapy represents a hope for many patients and is an undoubtful great step for the approach to this disease that has been neglected and misdiagnosed for a long time [13]. Indeed, recent data obtained via whole-genome and whole-exome sequence analyses together with biological samples screening [13] suggest that AADC deficiency is less ultra-rare than suspected. 

However, gene therapy has been applied taking into consideration the severity of the phenotype, irrespective of the genotype. This could lead to difficulties in interpreting the follow-up data, since the fully functioning AADC enzyme produced by the exogenous inoculation of its cDNA could be present concomitantly (or not) to endogenous AADC variant chains synthesized starting from the mutated gene. This would give rise to a complex AADC protein population.

**Figure 2:**
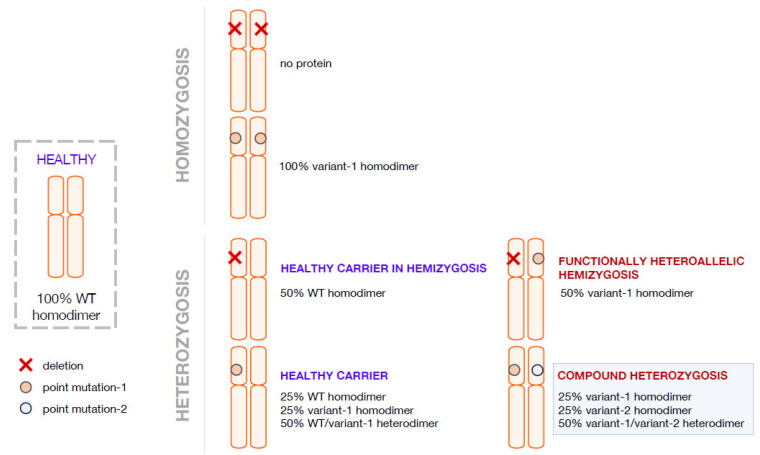
Possible combination of alleles in AADC deficiency in homozygosis and in heterozygosis. Representation of possible allele combination in healthy, homozygous and heterozygous conditions is drawn. In blue are the supposed healthy individuals or theoretical healthy carriers; in red, the affected individuals.

A contribution to the understanding of the severity/mildness of a phenotype arises from the characterization of the variant proteins aiming to determine the molecular cause responsible for the decreased activity of the variant species synthesized in the affected patients. The fact that a homozygote will produce only one type of AADC variant is more advantageous for structure-and-function correlation studies addressed to dissect the molecular basis for variant pathogenicity. Several papers have been focused on homodimeric AADC variants mimicking the AADC protein population of the homozygous patients, and an explanation for severe or mild clinical phenotype has been advanced and supported by structural and functional biochemical data, even if some inconsistencies have been observed and could be generally interpreted on the basis of individual genetic variability and environmental effects [14,15,16,17,18]. Overall, the investigation of protein machinery and its performance permits a reliable explanation of severity/mildness in homozygous AADC deficiency patients [17]. Interallelic heterozygosis determining functional hemizygosis arises when one allele leads to the synthesis of a variant polypeptide chain and the other is unable to do the same due to mutations in splicing sites or deletion. This leads to the production of only one type of variant enzyme, overlapping with what happens in homozygosis, unless in decreased amounts. Compound heterozygous patients would theoretically produce a mixed protein population with AADC homodimers and heterodimers (Figure 2). This complexity could prevent the dissection of the clinical phenotype. A further addition to complexity is the fact that it is unknown whether, in the presence of two alleles, there is a preference for homodimers or heterodimers or a combination thereof in mendelian terms. Moreover, protein folding or PLP binding and/or affinity complicate the situation from a predictive and mechanistic point of view.

The AADC enzymes produced by four AADC compound heterozygous patients have been characterized [4,19,20], revealing no apparent correlation between negative or positive complementation and clinical phenotype.

In this review, we focus on the AADC protein population present in pathogenic compound heterozygous and heterozygous AADC deficiency genotypes. The biochemical characterization of the protein species is examined regarding (i) the molecular basis for positive or negative complementation in compound heterozygosis from a protein point of view, (ii) the suggestions that in silico analyses can support the clinical interpretation, and (iii) the presence of AADC deficiency symptoms in heterozygous individuals, supposed to be healthy carriers. Overall, a rationale for mildness versus severe disorder expression could be advanced together with the need for a deeper screening of the population, with particular attention to the various components of the affected families, not only in genetical but also in biochemical terms.

## 2. Compound Heterozygosis and the Interpretation of the Clinical Phenotype on the Basis of the Combination of Genetics, Computational Methods, and Functional Analyses

Compound heterozygosity in rare diseases concurs in widening the spectrum of possible manifestations of these disorders and their genetic variability. Often, and also in AADC deficiency, it is the most frequent genotypic situation. One of the possible effects of the co-existence of two different alleles is positive or negative complementation, defined as the dominance effect of one allele over the other in terms of residual protein activity. In other terms, when the measured activity is higher or lower than the expected one (given by the average values of the individual alleles), a complementation phenomenon occurs. This could increase the complexity in the correlation of the genotype to the phenotype. In addition, since each mutated allele produces a variant polypeptide chain, if the functional protein is a monomer, the protein population will be a mixture of chains carrying exclusively one or the other alteration. Instead, in the case of a functional dimer or an oligomer of higher molecular size, the protein pattern increases its complexity due to multiple possible chain combinations. Combinatory calculations used in genetics to calculate the theoretical number of possible combinations (n!/r!(n − r)!, where n represents the number of genetic variants analyzed in a study and r represents the number of genetic variants per combination), do not entirely mirror the intricacy of polypeptide chain quaternary assembly, increased by the different geometrical arrangement of the subunits in such a level of protein organization. 

Reports in the literature concerning correlations among the compound heterozygous genotypes, the possible interallelic effects, and the metabolic and the clinical phenotype have been carried out for a few enzymes implied in monogenic diseases. 

In argininosuccinate lyase deficiency, the activity of the related homotetrameric enzyme was assayed by means of complementation assays performed with cultured fibroblasts of a certain number of patients, and results showed interallelic complementation effects [21]. Inherited defects in homodimeric methylmalonyl-coA mutase cause methylmalonic acidemia. Cell-free activity assays of the recombinant enzyme carrying the variants of a compound heterozygous patient could be interpreted on the basis of interallelic complementation [22]. The clinical correlation to the protein structural and functional features has been proposed by studying a larger number of protein variants carried by a significant group of patients [23]. The collected data of interallelic complementation were more predictive of the clinical outcome than the nature or the location of the mutation within the gene. Even if no genotype-to-phenotype correlation emerged, the potential of a designed therapy based on the structure/function of the variant protein was advanced [23]. A great number of papers have been published on the homotetrameric phenylalanine hydroxylase, aiming to correlate genotype to metabolic and clinical phenotype [24,25,26,27,28,29,30] resulting in phenylketonuria (PKU). Regarding compound heterozygosis in PKU, the dominance effects of one allele over the other leading to positive or negative complementation have been evaluated by measuring residual activity following expression of heteroallelic protein variants in eukaryotic systems, and an interpretation for complementation has been provided [25]. The correlation with the metabolic phenotype of patients was good, but some discrepancies appeared that were explained on the basis of epigenetic factors and differences in assaying activity in a cell model or in individuals, in addition to differences in protein folding and instability [25]. In order to find a predictive strategy, a method based on allelic phenotype value (APV) was calculated from the frequencies of metabolic phenotypes for hemizygous genotypes [30]. This parameter is a number with fixed different values (0, 5, and 10) attributed to individual variants present in hemizygous patients with classic PKU, mild PKU, and mild hyperphenylalaninemia, respectively. From computations, it is found that a variant with higher APV (mild) dominates over one with lower APV (severe). Attempts to explain this dominance in terms of protein population carried out by homozygous and heterozygous patients have been proposed using available bioinformatic programs of protein stability/damage prediction [29]. Genotype–phenotype associations were further validated by correlating APV with the metabolic phenotype [31] as well as with enzyme residual activity in vitro and protein expression in the case of classic PKU [29].

An integrated approach involving in vivo, in vitro, and in silico analyses was carried out for a variant of the homotetrameric succinic semialdehyde dehydrogenase (SSADH), whose deficiency causes a neurometabolic disorder caused by increased levels in g-amino butyric acid (GABA) and the accumulated toxic derivative g-hydroxybutyric acid (GHB). The metabolic and biochemical data were discussed in the light of the possible combinations of variant and native monomers in the quaternary structure assembly of the functional tetramer [32].

Nonketotic hyperglycinemia (NKH) or glycine encephalopathy is an inherited disease of glycine cleavage system governed by a four-enzyme complex, comprising glycine decarboxylase, a homodimeric PLP-dependent enzyme, playing a major role [33]. An integrated approach among clinical, biochemical, genetic, and computational analyses was applied on a cohort of patients in order to predict disease severity. A detailed number of factors (age of diagnosis, epilepsy among the initial symptoms, CSF/plasma glycine ratio) were considered and accompanied by information on protein stability and residual or null activity of the related homodimeric mild or severe variants proteins. These parameters were used as a scoring system for severity prediction and correlation of genotype to phenotype. The combination of two severe variants gives rise to a severe phenotype, while two mild variants lead to an attenuated phenotype, as expected. Interestingly, the presence of one severe with one mild variant resulted in a severe phenotype [33], in contrast to previous reports [34,35]. This was explained by the fact that in this combination, at least one variant has a complete loss of function in terms of protein activity.

The latest published data concerning enzymatic hetero-oligomers concern alanine glyoxylate aminotransferase, a homodimeric PLP-dependent peroxisomal enzyme whose deficit causes primary hyperoxaluria type I, leading to renal disease. A positive complementation has been reported for the heterodimeric S81L/G170R, whose variants’ combination was present in a compound heterozygous patient. Interestingly, both the homozygous G170R and the interallelic functionally hemizygote S81L develop renal disease, and these patients are more affected than the compound heterozygote [36]. Interpretation of these effects was provided by the dissection of the structural and functional features of the related homodimeric and heterodimeric proteins [36], based not only on measurements of activity but also, as was underlined, the level of expression, the affinity for the cofactor, the subcellular localization, and the ability to detoxify glyoxylate, which are important to evaluate to gain more in-depth information [36]. This investigation on a wider spectrum revealed that the characterization of recombinant dimeric proteins is an additional and reliable strategy to monitor how the alteration in signals other than activity measurement could be valuable for interpreting the extent of catalytic impairment (for higher oligomers, this is difficult due to the exponential increase in possible combinations of the two allelic variants). In this manner, the molecular basis for protein pathogenicity could be achieved. In the case of compound heterozygosity, the resulting homodimeric and heterodimeric variants could be expressed and characterized and the molecular basis for “enzymatic phenotype” unraveled. This needs to then be correlated to the genotype, either homo- and heterozygous, to ultimately understand the clinical phenotype. These investigations should give more strength and validation to the information given by computational programs such as FoldX, PoliPhen-2, Brenda, or SIFT algorithms that make predictions on enzyme stability and possible structural alterations and predict a potential output for the related effects on activity.

## 3. Compound Heterozygosis in AADC Deficiency

In the last few years, in an attempt to correlate AADC variants’ structural and functional features to the clinical phenotype, we carried out an extensive investigation of the protein population theoretically present in four compound heterozygous patients [4,19,20]. In addition to the homodimers theoretically produced by these patients, we also succeeded in expressing, producing, obtaining in good yields, and characterizing the heterodimers of each variant combination present in the patients. Results obtained with these heterodimeric species show that there is no apparent correlation between positive or negative interallelic complementation to the mildness/severity output of the clinical phenotype (Table 1). In order to determine the individual effect of each variant present in the heterodimeric protein, the correlated homodimers were expressed and purified and their structural and functional features measured [4,14,19,20] since they could play a substantial role in the protein constellation (theoretically 50% of the AADC species) of each affected compound heterozygous patient (Table 2). Unfortunately, only for two of these enzymatic variants was the homozygous patient identified, and the clinical phenotype correlates with the enzymatic features both for T69M and R347Q AADC variants [4,14].

Overall, the positive or negative complementation of a heterodimer (Table 1) does not seem to be related to the mild or severe effect on the activity of the less affected variant composing it, nor to the value of the catalytic constant *k_cat_*, nor to the affinity of the cofactor and, finally, nor to the clinical phenotype of the patient. 

**Figure 3:**
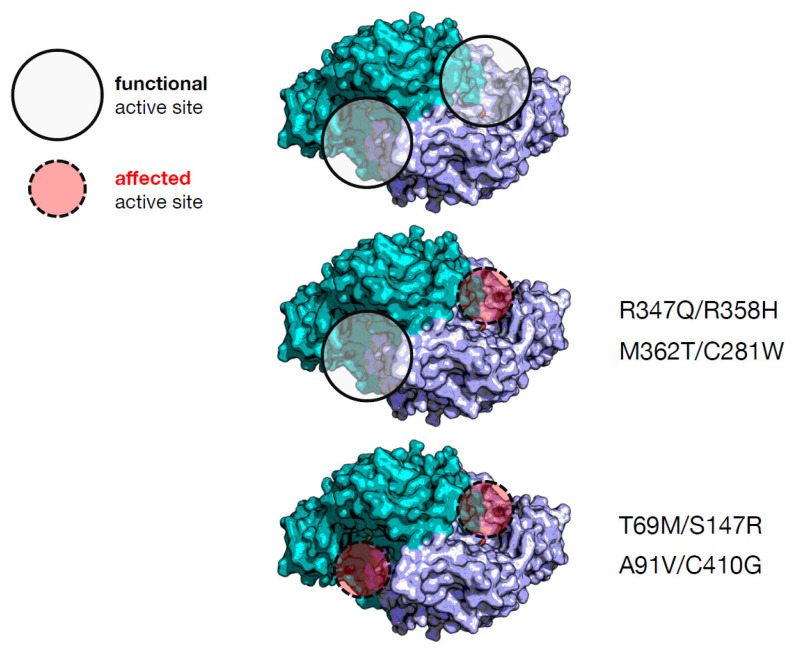
Possible effect on the active sites of AADC through combination of amino acid alterations in the heterodimers synthesized by compound heterozygous patients. The protein coordinates are taken from [37]. The two different colors refer to the functional AADC dimer displaying the antisymmetric arrangement. The circles are pointed on the active sites that could be altered or not by the amino acid alterations in the four heterodimers.

A rationale to predict the output of the interallelic complementation is the effect exerted by the single variants on both or only one active site [4]. Given the antisymmetric arrangement of the dimer [37] and the presence of loops protruding from one subunit to the other [38], each active site is composed by residues of both subunits. Following this view, the inspection of the crystal structure and bioinformatic analysis of in silico mutagenesis can predict that two severe homodimeric variants (R347Q and R358H) [14,19] could give rise to a heterodimeric combination where both amino acid substitutions affect the same active site, leaving the other site quite functioning to positively complement (Figure 3). On the other side, two mild ones (M362T and C281W) [4] affecting the same active site, as in M362T/C281W, could give rise to a negative complementation effect in activity, possibly due to the solubility problems of one of the subunits. The C281W homodimer could not be purified due to its high insolubility [4], a feature shared by other pathogenic variants of the same region, namely E283A [17] or R285W [14]. This could give rise to a protein with only one active site.

Instead, irrespective of severity or mildness of their homodimers (T69M, S147R, A91V and C410G) [4,14,15,20], the active sites of the related heterodimers (T69M/S147R, and A91V/C410G) are both affected, as revealed by the combination of bioinformatic inspection and kinetic assays, and thus, negative complementation takes place. A correlation between number of affected active sites and positive/negative complementation emerges for these four pathogenic AADC heterodimeric variants. However, as shown in Table 1, there is no correlation with the clinical phenotype. In order to find a predictive marker of clinical output, we observed that the catalytic parameter, *k_cat_*, for the heterodimeric proteins shows the following increasing trend: T69M/S147R, A91V/C410G, R347Q/R358H, and M362T/C281W. This tendency is not correlated to the values of the equilibrium dissociation constant for the PLP cofactor, which can be considered an indication of the degree of structural damage of the active site. T69M/S147R and R347Q/R358H are the two less structurally affected heterodimers in terms of PLP affinity, but the related compound heterozygous patients are the most affected. Since the C281W variant is poorly soluble and is presumed to have folding difficulties given the exposure of a substituted apolar residue at the place of a polar one [4], it can influence allele combination in patients and the relative abundance in a homodimer–heterodimer equilibrium. Overall, residual activity of recombinant protein variants as well as interallelic complementation could not correlate to the clinical phenotype, since other factors should be taken into consideration, such as protein folding, PLP binding, and the number of active sites compromised by amino acid substitutions. It is also evident that these observations are based on complete characterization of a limited number of genotypes, and more research should be carried out to reinforce interpretation and/or provide new suggestions.

Interestingly, our data agree with what was found by [33] in terms of clinical prediction since severity/mildness seems to be dictated (at least for dimeric enzymes) not by the mild substitution in terms of activity but by the combination of factors involving structural and functional elements of the active sites of the affected protein.

It would be worthwhile to find a strategy to precisely define mild, attenuated, and severe AADC deficiency in order to apply the APV algorithm that could be very informative. Meanwhile, a combination of studies on the recombinant enzyme species in solution and in silico as well as transfection into appropriate cell models of the variant cDNAs with multi-tag vectors could be an informative approach to predict phenotype and understand allele dominance.

## 4. Pathogenic Heterozygosity in AADC Deficiency

Heterozygous individuals in AADC deficiency are defined as healthy carriers and usually represent the parents of the affected patients. However, widespread reports in the literature deal with cases of heterozygosity associated with clinical signs. These are normally referred to as mild patients, but with some exceptions. In 2018, Portaro et al. [39] published the case of a woman affected by behavioral problems who was diagnosed in adulthood with S250F AADC heterozygosity and died before starting therapy. Since the phenotype in S250F homozygosis is attenuated [14,18], it is tricky to understand the reason for pathogenicity. The authors concluded that the symptoms were not so evident and only overlapped with those of AADC deficiency and that the presence of the mutation in heterozygosis alleviates clinical outputs. In addition, the paper shows that the patient possessed other mutations in other genes identified by next-generation sequence analyses, suggesting the relevance of the whole genetic inheritance. Another case was reported by [40] involving a AADC heterozygous individual (case II-2) that reached adulthood but presented with severe cerebral palsy and mental disability, microcephaly, and spastic–dystonic tetraparesis with dystonic scoliosis. The metabolic profile in plasma shows some values similar to those of the affected compound heterozygous siblings, such as homovanillic acid and 5-hydroxyindoleacetic acid (markers of affected aromatic amino acids L-Dopa and L-5-hydroxytryptophan imbalance), but activity in plasma was about half that of both her parents (one of whom with her identical genotype in the gene for AADC) (Table 1 in [40]). These results were not commented on but indicate that heterozygous carriers could also show some disease signs. 

Finally, the Taiwan list of patients published by [41] shows the presence, among others, of patient number 37 (Table 1 in [41]) who is heterozygous for AADC carrying the splicing mutation C.714 + 4A > T on one allele. This mutation is the result of a typical founder effect and is very severe both in homozygosis and in heterozygosis [2,3,41]. Patient 37 presents with a mild phenotype possibly due to compensation by the WT allele, leading to a protein pool with only WT AADC, unless decreased in amounts.

This implies on one hand the importance of a registry comprising not only the affected patients but also the natural history of the parents, siblings, and the whole families, as the International Working Group on Neurotransmitter Related Diseases (iNTD) registry aims to collect (https://www.intd-registry.org/ accessed on 30 August 2022). On the other hand, caution should be taken in considering all heterozygous individuals as healthy carriers. To researchers, it would be worthwhile to compare CSF metabolic profiles and plasma AADC activity of a large familiar “bucket”; to clinicians, to be aware of psychiatric, depression, and behavioral problems.

In addition, the comparison of the genomic data not confined to AADC but to other possible variants in other proteins for the enlarged family could offer some hints for possible interplay between AADC and other factors.

## 5. Conclusions

Complexity in predicting the clinical phenotype in AADC deficiency patients could be also tackled by studying the structural and functional features of the protein population hypothetically synthesized by compound heterozygotes. This could provide us with sufficient knowledge to integrate genetic analyses and prediction computational tools. The presence of a heterodimer, in addition to the homodimeric AADC variants, and the fact that its amino acid substitutions exert their effects in trans, given the antisymmetric quaternary structure architecture, could influence in different manners the two active sites of the heterodimer. Bioinformatic analyses using the crystal structure of the dimeric AADC are the rational basis for predictions in terms of enzyme stability and activity that could be validated in solution with recombinant enzymes and, hopefully, in a more reliable neuronal cell model. By correlating all data concerning the localization on a particular domain of the protein, the solubility of the recombinant variant, the structural integrity measured by secondary and tertiary structure signals, the PLP affinity and the catalytic parameter determination and modeling the amino acid substitutions on the crystal structure by bioinformatic visualization, in silico mutagenesis, and energy minimization, it could be possible to advance precise prediction on the molecular basis for enzyme functioning. Further steps in compound heterozygotes should take into consideration the data obtained with homodimers and heterodimer AADC variants, taking into account the whole protein population and analyzing them in an integrated manner.

Particular caution should be taken for heterozygotes still considered healthy carriers since scattered reports reveal that they can manifest a mild or severe clinical phenotype. Thus, the concept that heterozygosity means a healthy carrier could be misleading for dimeric and oligomeric enzymes, since the WT allele could not necessarily compensate for the mutated one. More investigation on these individuals and on their protein composition and effects is thus desirable since their protein species could provide insight into the allele dominance of selected variants with respect to the WT allele. In addition, deeper knowledge of familiar history regarding even minor motor or developmental delays or psychotic disorders or unexplainable neurologic episodes is desirable to draw a complete picture of this multifaceted neurotransmitter disease.

## Figures and Tables

**Table 1:** Characteristics of the heterodimeric AADC proteins present in four compound heterozygous AADC deficiency patients.

Heterodimer	Individual Effect on Activity	Interallelic Complementation in Activity	Number of Active Sites Affected	Catalytic Constant *k_cat_* (s^−1^) ^d^	PLP Binding Affinity (nM) ^d^	Clinical Phenotype Output
T69M/S147R ^a^	Mild/severe	negative	2	0.27 (4%) ^e^	310 (3.9-fold)	severe
M362T/C281W ^a^	Mild/mild	negative	1	1.8 (29%)	1232 (15.4-fold)	mild
A91V/C410G ^b^	Severe/mild	negative	2	0.38 (6%)	≈000 (≈12.5-fold)	mild
R347Q/R358H ^c^	Severe/severe	positive	1	0.45 (7%)	360 (4.5-fold)	severe

^a^ Data were taken from [4]. ^b^ Data were taken from [20]. ^c^ Data were taken from [19]. ^d^ Data for double tagged wild-type (WT) AADC measured under the same experimental conditions were taken from [4] and are *k_cat_* = 6.3 ± 0.1 s^−1^ and equilibrium PLP binding affinity K_D_ = 80 ± 20 nM. ^e^ Percentage with respect to WT AADC. ^f^ Fold with respect to WT AADC.

**Table 2:** Characteristics of the homodimeric AADC proteins present in four compound heterozygous AADC deficiency patients.

Homodimer	Expression ^a^	Catalytic Constant *k_cat_* (s^−1^) ^b^ per Dimer	PLP Binding Affinity (nM) ^b^	Homozygous Patient Phenotype
T69M	good	3.5	100	mild
S147R	quite good	0.0090	846 ^c^	n.p.
M362T	good	4.6	271	n.p.
C281W	poor	n.d.	n.d.	n.p.
A91V	quite good	0.0083	782	n.p.
C410G	good	4.4	1070	n.p.
R347Q	good	0.087	54	severe
R358H	good	0.030	1300	n.p.

^a^ With respect to the WT AADC. ^b^ Data of his-tagged WT AADC measured under the same experimental conditions were taken from [4,14] and are *k_cat_* = 7.6 ± 0.1 s^−1^ and equilibrium PLP binding affinity K_D_ = 100 ± 10 nM. ^c^ [15]. n.d., not determined because of the insolubility of the variant [4] and the poor rescue in the soluble fraction. n.p., not present in homozygosity.

## Data Availability

Not applicable.

## Abbreviations

Aromatic amino acid decarboxylase, AADC; pyridoxal 5′-phosphate, PLP; cerebrospinal fluid, CSF; 3-*O*-methyldopa, 3OMD; phenylketonuria, PKU; allelic phenotype value, APV; succinic semialdehyde dehydrogenase, SSADH; g-amino butyric acid, GABA; g-hydroxybutyric acid, GHB; nonketotic hyperglycinemia, NKH; International Working Group on Neurotransmitter Related Diseases, iNTD.
